# Psycho-Socio-Cultural Determinants of Food Choice: A Qualitative Study on Adults in Social and Cultural Context of Iran

**Published:** 2017-10

**Authors:** Arezoo Haghighian Roudsari, Abouali Vedadhir, Parisa Amiri, Naser Kalantari, Nasrin Omidvar, Hassan Eini-Zinab, Seyed Mohammad Hani Sadati

**Affiliations:** 1 Department of Community Nutrition, National Nutrition and Food Technology Research Institute, School of Nutrition Sciences and Food Technology, Shahid Beheshti University of Medical Sciences, Tehran, Iran.; 2 Department of Anthropology, Faculty of Social Sciences, Tehran University, Tehran, Iran.; 3 Department of Social Determinants of Health, Research Institute of Endocrine Sciences, Shahid Beheshti University of Medical Sciences, Tehran, Iran.; 4 Department of Integrated Studies in Education, Faculty of Education, McGill University, Montreal, CANADA.

**Keywords:** *Food Choice*, *Psychological*, *Social*, *Cultural*, *Grounded Theory Methods*

## Abstract

**Objective:** Food choice is a process through which people think, feel, and eat food. It does not only influence individuals' health and well-being, families and communities, but also it has an effect on regional, national, and global levels. This qualitative study was conducted to explore perceptions and lived experiences of Tehran adult residents on psychological, social and cultural determinants of food choice.

**Method:** In this qualitative design, we recruited 33 adults aged 30 to 64 years from various districts of Tehran, capital of Iran, and we explored how people make decisions about food choice in practice and shape their perception, attitude, and eating practices. An individual in-depth semi-structured interview guide included major questions with follow-up probes was used to explore participants’ current and past eating habits from childhood to adulthood, dietary change at different life courses, and effective psychological state on food selection in different seasons.

**Results:** This study revealed that food choice in the studied adults (30-64 years old) was widely influenced by psychological, social, and cultural determinants, which can be categorized into 5 main themes: cultural context and patterns; social Structure and norms; information resources and media; household and family structure; and nutrition transition.

**Conclusion:** The findings clarified the importance of social and cultural contexts, which influence the food choice of adults in a metropolis like Tehran. Many of these concepts are contextualized from childhood. These findings could serve as guideline to design socio-culturally appropriate strategies and improve dietary behaviors of Iranians.

Food and food choice are central components of typical and modern approaches and policies for managing health problems including diabetes, coronary heart diseases, and other food-related diseases (1). As defined by Food Standards Agency (FSA), food choice is "the selection of foods for consumption, which results from the competing, reinforcing, and interacting influences of a variety of factors." These range from sensory, physiological, and psychological responses of individual consumers to interactions between social, environmental, and economic influences including the variety of foods and food industry activities to promote them (2).

Food choice not only influences health and well-being of individuals, families, and communities, but also influences agriculture, environment, business, culture, and economy at local, regional, national, and global levels (3). These efforts are not limited to one branch of social sciences, and a variety of disciplines are involved to clarify this process (4). Past practice is a consistent predictor of current practice. Individuals’ past food choices, thoughts, and feelings associated with those choices and the changing temporal, social, and historical contexts that shape those choices all make up their life course paths of food choice (5).

People construct their food choices, and life course perspective explains how this construction occurs (6). This perspective is made of multiple notions to understand people's thoughts on the food choice including trajectories, transitions/events, cultural and contextual influences, timing in lives, and adaptive strategies. It recommends 3 frameworks for understanding food choice: temporal, social, and historical (5, 7).

Sociocultural variables, among these factors contribute to food selection and eating practices affecting the purchasing behaviors of individuals and consist of ethnicity, religion, social class, reference group, family, and demographics including age, sex, education, occupation, income, marital status, and geography mediated by individuals’ attitudes and beliefs (8). Some studies have been conducted on factors influencing food choice and revealed the significant effects of sociocultural determinants on selecting variety of foods worldwide (5, 9-17).

The developing countries in Middle East including Iran are in the process of nutrition transition and prevalence of obesity-related diseases including cardiovascular disease, Type 2 diabetes, hypertension, and cancers (18-19). In Iran, nutrition transition is taking place with accelerated demographic change, urbanization, and social expansion in spite of insignificant economic growth. Although the pattern and quality of consumed foods largely depend on households’ income and food selection, particularly in people with higher income, it does not entirely depend on economic forces (20). A study on Iranian households’ food intake revealed that low nutrient density diet is not only an income-driven issue and in upper 50th percentiles of income, nutrient requirements are met at energy levels of 3000 kcal per day (21). Thus, understanding how food choices have changed across low- and middle-income nations is a priority for healthy eating promotion (22).

Little research has been conducted on the cultural meaning, showing food choice process of Iranians such as snack consumption (23), healthy eating barriers (24-25), and fruits and vegetables intake in adolescents (26), most of which have emphasized on one of the food group intakes or dietary habits. Thus, it was highly important to conduct a comprehensive research to understand the cultural, social, and psychological aspects that affect people's food choice. Psycho-socio-cultural determinants of food choice have been considered less frequently in the past studies, and a qualitative research is apt to achieve this purpose. Thus, the current qualitative study was conducted to explore perceptions and lived experiences of adults living in Tehran on psycho-socio-cultural determinants of food choice. This was the first qualitative study in Iran to explain adult participants’ points of view, which may valuably contribute to identification of unknown food choice determinants.

## Materials and Methods

This qualitative study explored how people make decisions on food choice in practice and how their perceptions, attitudes, and eating practices are shaped (27). A typical grounded theory method (Strauss and Corbin's Approach) was applied to iteratively collect and analyze the data to study any interactional practice and effective component in shaping food choice (28). 

Setting, Participants, and Data Collection 

During April 2014 and March 2015, 33 adults aged 30 to 64 years were recruited from various districts of Tehran, capital of Iran, with a population rate of 11 690 000 using purposive sampling (29) to identify and select the information-rich individuals, especially knowledgeable or experienced on the matter (30). In addition, individuals were selected based on their availability and willingness to participate and the ability to communicate their experiences.

An in-depth semi-structured interview guide including major questions with follow-up probes was used to explore participants’ current and past eating habits from childhood to adulthood, dietary change at different life courses, and effective psychological state on eating and food selection in different seasons (Table 1).

Interviews lasted 30 to 45 minutes and were conducted by the chief researcher in a private place. The interviews were audio-recorded and transcribed word by word. Participants filled a demographic questionnaire on gender, age, occupation, marital status, years of education, household type and dimension, residence areas, and ethnicity. Interviews consisted of open-ended questions, and the participants were encouraged to talk about their thoughts. Important field notes, nonverbal reactions of the participants, and memos were recorded during the interviews. Sampling continued until data saturation, i.e., later informants did not add new perceptions (28). Sociodemographic characteristics are presented in Table 2.

***Data Management and Analysis***

Data were analyzed using constant comparative analysis. Data collection and data analysis were performed simultaneously, considering the basic principles of grounded theory method. The process of data analysis was performed applying open, axial, and selective coding stages proposed by the mentioned approach (28).

Interview transcriptions were reviewed and imported into the qualitative software, MAXQDA 11, for open and axial coding (31). Participants' key statements were identified, open codes and similar codes in meaning were classified into subcategories. In axial coding, based on the proximity of their purports, subcategories were grouped into categories. Main themes emerged by integrating the categories with due attention to their properties.

***Ethical Considerations***

The study protocol was approved by the ethical committee and Research Council of National Nutrition and Food Technology Research Institute (ECRC-NNFTRI) in accordance with the guidelines of Iranian Ministry of Health and Medical Education (approval number: 450/1762; approved on 15/11/2014).The study objectives were clarified for the participants and informed consents were obtained for recording the interviews.

Quality, Data Trustworthiness, and Rigor of the Study

To improve the credibility of the study, those who had plenty of knowledge and imagination about population policies in Iran were recruited. Diversity in recruiting the informants gives more credibility to data (selecting participants with different approaches and experiences from different governmental, legislative, non-governmental, academic, and international institutions). Triangulation was used in all research phases including the types of data and collection methods (interview, observation, memoing, and documents) to gather the viewpoints of key informants and documents, and achieving rich results. All interviews were immediately and fully transcribed, documented, and sent to the participants to collect their opinions about the coherency, integrity, and comprehensiveness of the text. Constant attendance of the interviewer in all interviews and spending enough time on gathering accurate and rich data, and clarity of methodology added to the transformability of the study. All interviews were performed by the first author to prevent bias and improve the accuracy through coordination within the framework of interviews, understanding the questions, answers, and perspectives of the participants. 

To ensure data trustworthiness, Lincoln and Guba criteria were used (29). Truth value of the findings was reinforced through recoding almost 40% of the interviews by a nutritional sociologist to compare the recoded results with the main emerged codes. Emerged understanding of the data was also debriefed and challenged with other team members to omit potential problems. To confirm transferability and fitness, thick description and purposive sampling were conducted; it was also sought by presenting as much detail as possible on procedures, decisions, and the context in which the research was conducted, enabling readers to evaluate the appropriate contexts to transfer the findings. 

## Results

According to the participants, the sociocultural determinants are the contextual factors in shaping individual perceptions of food choice processes and configuring attitudes, habits, practices, and discourses in relation to food choices from childhood to adulthood. Food choice does not occur in a vacuum and people make decisions based on their psycho-socio-cultural backgrounds, and they constantly try to equilibrate among these factors, personal disposition, and what they learn throughout their lifespan about food and eating practice from significant and influential individuals including parents, family members, peers, friends, partners, and couples. They link the past experiences with life lessons to compile current food choices, which might have had some turning points in the life course.

***Main Themes and Categories***

A closer look at the findings reveals that psycho-socio-cultural determinants of food choice in 30 to 64-year old adults can be categorized into 5 main themes: cultural context and patterns; social structure and norms; information resources and media; family structure; and transition in nutrition. 

**Theme 1: Cultural Context and Patterns**

*•* *Cultural meanings and perceptions of cookery*

Some participants believed that instead of spending time on cooking, they can have more time for other priorities of life. This kind of thinking is due to lack of sufficient cooking skills central to preparation of variety of foods. Also, an educated female declared, 

“I had no academic and systematic training in cooking. In fact, I imitated all about cooking from my mom or my mother-in-law.) (a 44-year- old female).

In fact, many women, especially those who were employed, were not interested in spending their time in the kitchen. One of the participants expressed,

“I sometimes do not have patience for cooking. I love everything except cooking at home. I like to eat stuffs at home ….” (a 43- year-old female).

Some participants stated that their feelings about cooking depended on the subculture or atmosphere of the family and parents' attitude towards cooking. In this view, some pointed out their mothers’ roles and beliefs about cooking. An educated female stated, 

“When I cook some new dish, my children like it. When I cook pizza with them, they also enjoy. I am interested in cooking and my son loves to cook.” (a 40-year-old female).

*•* *Understanding of traditional and local foods*

Based on some participants' quotes, indigenous, natural and traditional foods are better and healthier compared with fast food and have a good impact on health status of people. According to a faculty member,

“If you eat an Iranian food, you won't overtake kidney stones, and all these take place with respect to the traditional knowledge or ethno-science.” (a 62-year-old male).

However, a number of participants addressed some challenges in preparing traditional foods in Iran, which included the lack of efficient proficiency, low interest of children, lack of suitable circumstances in living place, and lack of the necessary ingredients. A participant claimed,

“When we want to add some indigenous foods into the family menu, it is in contrast with the tastes of children and living style in a metropolis like Tehran.” (a 44-year-old female).

**Table 1:** Interview Guide Protocol to Conduct the Qualitative Interview

**Main questions**	**Probes**
1. Please explain about all kinds of foods that you consumed daily.	- Consumed foods at weekend and during the week - Snacks - Dietary habits
2. What are your criteria for selecting foods?	
3. How much do you eat and what do you eat when you are happy, sad or angry?	
4. What is your opinion about Halal and Haram foods?*	
5. Please tell us about your parents' dietary habits.	- Method to prepare foods - Food resources
6. Has your dietary habits changed since childhood?	- If your answer is yes, please clarify the time and kind of changes.
7. What are the reasons of dietary habit changes?	
8. What are the factors induced to not choose your favorite foods?	
9. If the condition is desirable, will there be changes in your choices?	
10. What are your food choices in special occasions like Ramadan?	- Kinds of foods - Amounts of foods
11. How are your food choices in various seasons?	- Kinds of foods - The reasons of selections
12. Is there any person whose opinion is important for you to choose foods?	- Who and what
13. How are your food choices in various situations in terms of time and place?	- Regularity - Place and persons which are with you
14. What is your opinion about local and traditional foods?	

* Forbidden or non-forbidden foods in Islam

**Table 2:** Sociodemographic Characteristics of the Participants who take part in qualitative study (n=33)

	**Frequency (%)**
**Gender** Female Male	22 (66) 11 (34)
**Age (year)** 30-49 50-64	21 (64) 12 (36)
**Education** Primary National Diploma Higher Education	10 (30) 6 (18) 17 (52)
**Marital status** Married Single Divorced Widowed	30 (91) 1 (3) 2 (6) 0 (0)
**Employment Status** Employed Housewife Retired	20 (61) 11 (33) 2 (6)
**Type of Household** 1 adult 2 adults 1 adult with children 2 adults with children	- 1 (3) 2 (6) 30 (91)
**Residence Area ** North South Center	11 (34) 6 (18) 16 (48)

**Figure 1:**
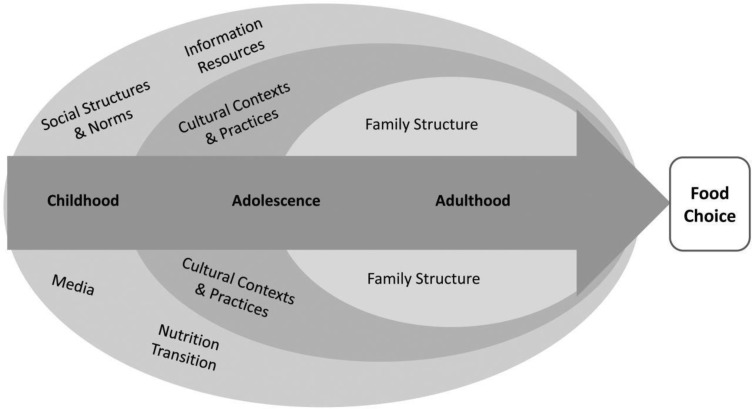
Conceptual Framework of Psycho-socio-cultural Determinants of Food Choice

*•* *Inspiration from traditional medicine*

A number of participants also considered the contribution of traditional and alternative medicine (TAM) to the process of food choice. According to them, TAM had positive effects on health status of people and contributed to their peace, relaxation, and comfort. They, implicitly or explicitly, pointed to the benefits of TAM, discussing on a kind of humor theory in the process of food choice. As a participant explained, 

“We know our body’s humor and choose our food according to it. In this way, our soul and body will be healthier and ready for daily activities.” (a 39-year-old female).

*•* *Religious beliefs and decrees*

Many respondents expressed the significance of a religious principal; namely, "Halal" in Muslim societies. They pointed out the undeniable significance of the notion of “Halal” in their everyday life and food choice process. Most participants emphasized “Halal” production and consumption of food. They declared that living in a Muslim country like Iran makes one typically not to get worried about religious slaughter.

“I have never thought about this issue as we live in Iran as a Muslim Shia country; all foods are Halal. However, when I go to non-Muslim countries, I have to take this issue into account.” (a 40-year-old female).

The participants described other religious parameters that affected the type and quality of foods selected including religious ceremonies and events like Ramadan and Muharram mourning that normally change the eating and food choice processes, which mainly enter a series of specific foods to the diet of people or mandatory choices that people would prefer to consume. A participant explained,

“In the holy month of Ramadan, we typically eat “Aash” [a traditional thick soup, which contains legumes, leafy vegetables, and noodles], while in mourning ceremonies of Muharram, we eat foods outdoors.” (a 39-year-old female). 

**Theme 2: Social Structure and Norms**

*•* *Accessibility of resources and facilities*

Easy access to resources and facilities were mentioned as a leading factor influencing food choices. The participants believed that having enough time intervenes with the process of decision- making regarding food choice. Time constraints affect purchasing, transferring, and preparing the desired foods. Sometimes, they were forced to choose foods compatible with their interests due to these limitations and only met the needs and interests of their family members. A young married woman shared her experience, 

“For me, availability of food is important because usually I do not have plenty of time to go out and buy stuffs.” (a 30-year-old female). 

In some cases, the participants were concerned about choosing healthy foods; however, they had to avoid them due to lack of enough time. 

“Fish needs to be cleaned and it takes a long time to prepare it. Honestly, I don’t have time to cook these kinds of food, and I prefer to ignore them.” (a 43-year-old female).

*•* *Job-related food constraints*

Another influencing factor reported by the participants was job conflicts. According to the participants, their job and working hours are not attuned with a healthy eating timing during the day, and consequently, they are forced to eat fast foods. Occupation and its constraints have a great impact on their food choices. They consume at least 1 or 2 main meals in the workplace and face problems such as low quality of foods offered in the workplace and lack of enough time for preparing their favorite foods because of women's occupation and irregularities in the eating time.

“It is very difficult to take food with me. Sometimes, I eat chocolate or biscuits or junk food for lunch.” (a 35-year-old female).

*•* *Social relationships*

For the participants, social relationship is also an important factor in choosing foods. The most influential factors were parties and gatherings, feasts and special occasions, holidays, and traveling, and also significant others, whose opinions are valuable to them. Food choices differ in quality and quantity based on the situation. For example, the food types consumed in parties are more diverse and contain more protein and oil, salads, vegetables, and beverages. As a senior woman expressed, 

“When we have guests, I have to prepare more foods, at least 2, to respect and honor my guests.” (a 52-year-old female).

In feasts and special events, special foods are consumed which could be ethno-culturally different. Eating practices in these events might be different from the daily and routine food consumption habits. 

“We eat sea foods, rice, and vegetables in the Persian new year (Nowrouz) or at the dawn of the New Year. Although we eat all these foods throughout the year, but it is different, as it is indeed a unique opportunity for families to come together.” (a 62-year-old male). 

During the holidays and travelling time, it is common to enjoy the opportunities to choose food considering the individual and family’s desire. In general, food choices are more comprehensive in holidays compared to other times because people have enough time for shopping, preparing and cooking food, and spending time with their family members. The participants described these foods as more diverse and complete. As a senior woman declared,

“Weekends are wonderful because we meet each other and do our best to have the best foods.” (a 62-year-old female).

The participants also pointed out the role of other significant people in social relationships and food choices. A woman explained, 

“I don't typically drink soda and coffee. When I am happy or I am with a lovely person, I enjoy eating everything including foods, sweets, and soda ….” (a 39-year-old female). 

*•* *Stigmatization of obesity and unfitness*

For many participants, food choice is affected by concerns about obesity and loss of fitness. Sometimes, they choose foods that can improve their appearance and avoid foods such as sweets, which are harmful for their fitness. General perception about obesity has caused a large number of people to get constantly worried about their size and weight. A male said,

“When I buy meat, I try to choose low fat beef or low fat dairy products. Well, obesity is now an undeniable health problem.” (a 38-year-old male).

**Theme 3: Information Resources and Media**

*•* *Nutritional education*

A large number of participants stressed that the nutritional knowledge of people plays an important role in identifying and choosing healthy foods. People's knowledge about foods properties, food preparation skills, foods, and health links may influence the food choice process. This kind of knowledge is derived commonly from educational background, training courses, type of readings, and occupation-induced information. A senior lady explained,

“We now have sufficient knowledge about healthy foods a, which we gained from mass media and reading variety of books and attending many classes; hence, we cook foods with illuminative views.” (a 62 year-old female).

*•* *Media and advertisement*

The participants are exposed to broad advertisements of manufactures, exporters, importers, and internet networks. Most of the participants believed that foods advertised by the mass media including TV programs and radio are safe, healthy, and harmless. They also pointed out the role of the internet as an important source for acquiring health and food-related information. Participants expressed these media somehow contribute to familiarity of people with valid brands and goods, 

“I try to choose foods with best quality and valid and trustable brand. These foods are commonly advertised in the media.” (a 32 year-old male). 

**Theme 4: Family Structure**

*•* *Socioeconomic Position (SEP) of household*

One of the crucial assertions of the participants was on the potential effects of SEP of individuals and households on the food choice processes including the quality, quantity, and the variety of foods selected. In this view, food choice is different in favorable and unfavorable SEP. And giving voice to the quality and values of food and its impact on health possibly takes place in favorable SEP. In some cases, overeating was common among a number of participants. For example, a higher educated participant described his overeating on the weekend in this way,

“I eat more rice on the weekend and I sometimes feel my weight increases in the beginning of the week.” (a 43-year-old male). 

There was an interest, in some cases, in people with higher education to consume traditional foods, but they had no sufficient skill to prepare them. Hence, they preferred to buy these foods from the market. As a participant declared,

“Some traditional foods are not made in our home, as its preparation is difficult and time-consuming; however, I try to buy these stuffs from healthy shopping and food centers.” (a 62-year-old male). 

In contrast, participants with unfavorable SEP reacted to these conditions differently from people belonging to the upper classes. They highlighted the role of the main meal eating pattern and tried to choose easily available foods that met their daily needs. They preferred foods that satisfied them and removed their hunger. These participants took strategies such as reducing the quantity and quality of foods, preparing some basic foods from their hometown cities because of cheaper prices, avoiding expensive foods and withdrawing other living costs to provide food costs to manage challenges of the food choice process.

“When I can't buy any foods, I have to buy less. If a food is expensive, I try to ignore it as my husband is just a simple worker. We are also tenants and have to pay a lot as rent.” (a 46 year-old female). F 

*•* *Role of the family members*

Most of the male participants pointed out that food choice depended on women's occupation and their choices, as the time they could spend for preparing food determined the food choices of the family. Each family member has a role in shaping health practices of other members. It seems that mothers’ knowledge and women's roles are meaningful.

“As women are usually responsible for cooking foods in our culture, regardless of the fact that they work out of home or not, they prepare foods for all members of the family. As a result, they do their responsibility according to their time.” (a 62 year-old male). 

**Theme 5: Transition in Nutrition**

*•* *Wide currency of the fast and outdoor foods*

Fast food consumption or eating foods at restaurants is the issue described by the participants. As they explained, this way of supplying foods is reasonable, easy, and fast. Individuals’ tastes and food preferences are getting transformed worldwide including in Iran, in line with social developments and new cultural patterns. For example, according to the participants' remarks, traditional foods are being replaced by new and fast foods that are apparently symbols of modernity. For people who are away from home, fast food is the shortest way to remove their hunger. As a woman put, 

“We all know that the culture of fast food consumption is not good; however, it is popular among people due to its cheap price and fast preparing process.” (a 39 year-old ).

For most participants, eating fast foods is an entertainment and may fill their leisure time, particularly among youngsters. As a participant explained,

“For many people, eating fast foods with the family is a reasonable way to spend leisure time.” (a 43 year-old male). 

*•* *Changes in dietary patterns*

Rapid changes in dietary patterns are central to the process of nutrition transition worldwide. The prevalence of this kind of thinking implicitly or explicitly accelerated the westernization and McDonidization of the Iranian society (31). As a participant stated,

 “Our society is becoming westernized. In line with these macro changes, westernization of our foods is inevitable.” (a 64 year-old male). 

In this view, some participants did not pay attention to problems of fast foods regardless of all warning messages of mass media and nutritionists’ health-related claims. They selected and consumed these foods not considering their possible negative effects on their health status. Turning points in lifespan regarding dietary patterns 

Turning points of participants’ life had led them to change the process of food choices or put it on the new track. Major life events such as getting married, getting and changing jobs, going to university, doing military service, and childbearing were the most important turning points of life courses of the participants in this study. As a male participant described,

“At the military service, we used to eat our meals on time and change our eating practices. After marriage, some changes occurred that have continued up to now.” (a 38 year-old male). 

On the whole, sociocultural determinants shape people's food choices during their lifespan, which are affected by many factors (Figure 1).

## Discussion

We explored 5 main themes including cultural context and patterns, social structure and norms, information resources and media, family structure, and transition in nutrition regarding sociocultural determinants of food choice. Findings revealed the complexity of food choices in psycho-socio-cultural determinants using the grounded theory method to deeply achieve participants' thoughts, feelings, and views. Cultural contexts and practices, social structures and norms, information resources and media, family structure, and transition in nutrition were the crucial factors influencing participants' food choices. The results of these determinants leading people to select their foods were in agreement with the findings of previous studies, which sought the wide range of agents and processes such as food choices of people (10-11, 13-14, 17, 33-36).

Accessibility of resources and facilities were another concept emerged by many researches on food choice (5, 9-10, 15, 17). Ease of access to foods, shopping, and food preparation were the participants' concerns about food as observed in Tehran residents by Farahmand et al. (24-25).

Despite these statements, they focused on the time constraints to prepare and eat variety of foods, especially in families that women worked, as observed in another study (12). Job limitation might make some changes in eating practices due to the lack of enough time to eat foods in workplace and poor quality of foods prepared there (5, 12).

Social relations have been considered in many studies as a determinant of individual food choices (10-11, 13, 16). Social communication with other people, coworkers, and friends made some modifications in food selection (5). In the social context, people attended some parties, feasts, and ceremonies influencing their eating behaviors and choices. There is evidence that eating with others can lead to eating more food compared with eating alone (37). 

In recent decades, changes have occurred in thinking ways with respect to body image and obesity in developing countries including Iran. According to Erving Goffman, body management is the basis for social relations (38). This thought is grounded in an individual's body image and behaviors or practices. The results of one study revealed that the prevalence of undesirable body image, especially among women in which modernity, awareness about globalization, lifestyle, negotiation in family, cultural capital, and marriage status on the whole could account for about 40% of women's body image in Iran (39). As asserted by the participants, their concerns about fitness could influence their food choice, as food avoidance or skipping meals sometimes referred to as a reason for fear of obesity, which is in accordance with previous studies (17, 40).

Farahmand et al. revealed that food preferences among Iranian families are shaped mainly by their children’s food desires (24) that echo the statements of study participants. A number of researches among new couples have revealed that ideals of shared eating lead to dietary convergence, balanced by food individualism (40); the current findings showed the prominent role of the mate for women and men. Lake et al. also indicated similar results in a longitudinal (ASH30) study (12).

The cultural practices of family and friends at special celebrations and holidays, in particular, provided occasions to eat culturally or ethnically determined foods and reinforce the importance of these foods. This study explored a wide variety of concepts rooted in the culture of Iranian community. The most important statements covered cultural meanings and participants’ thought regarding the concepts of cookery, local foods, traditional medicines related to types of foods and religious decrees such as consideration of “Halal” foods that determined the type, amount, and style of eating. All the mentioned aspects were culturally-specific practices, which highlighted the Iranian cultural context that has to be considered in the studies on eating behaviors changes. 

As observed in other studies (12, 24-25, 41), the present study indicated the vital role of education on the quality and quantity of food choices. They acquired their nutrition related knowledge from some channels such as educational courses, occupation-induced information, books about food and nutrition, newspaper articles, magazines, and television programs, which influenced their food selections. There is much evidence demonstrating the influences of marketing activities on food selections (42-43); likewise, the participants believed that foods advertised by TV, radio, and the internet were safe, healthy, and reliable for consumption.

As demonstrated in the literature, the price of food is a determinant in food choices (5, 13, 17, 24, 44-45). It was also proved, in the current work, as a significant factor in choosing foods. Depending on the socioeconomic position of the participants, income level of household indicated the amount of money spent for food purchase. Similar to another research by Mancino et al., low-income individuals economized through selecting more discounted items and low- quality foods compared to high-income participants, who chose high quality and healthy foods (46). 

Diets in the 1970s began to move towards increased reliance upon processed foods and away from home intake and greater use of edible oils and sugar-sweetened drinks (22). There was a significant imbalance in food consumption and low nutrient density characterizing diets at all income levels, overconsumption among more than a third of households and food insecurity among 20% of Iranian population (20). The results of a study on Tehran adult residents revealed that they needed diet improvement (47). Another study revealed an unfavorable trend towards a westernized diet in West Asia and in North Africa (48). As outlined in the literature, the participants in this research study stated the unavoidable roles of outdoor and fast foods in their food choices. This was especially emphasized among younger adults as an entertainment when spending time with their friends, coworkers, and families. Most of them declared shifting from homemade foods to store-bought, low-cost, and easy prepared foods. 

In the meantime, they referred to the significant turning points in their lifespan, which induced some changes in the path of their choices. These were explained by participants as effective times to modify the quantity, quality, and style of their eating. They described different experiences and periods in their lives such as childhood, adolescence, adulthood, parenthood, work, military service, pregnancy, and marriage, which are parallel to other studies (10, 14, 24, 40).

## Limitations

In terms of study strength and limitations, the qualitative methodology employed allowed to deeply explore these determinants in detail. The participants were purposively selected from various regions of Tehran with different SEP, occupation, and educational levels. This was a prominent study, which explored wide ranges of sociocultural determinants of food choices among Iranian community. One of the main limitations was that participants were selected from the urban community and did not represent the rural areas. Thus, in future investigations, it is recommended to gather data from these areas to make possible the generalization of the findings.

## Conclusion

This study confirmed the importance of psychological, social, and cultural contexts influencing the food choice of adults in the metropolis of Tehran. Many of these concepts are contextualized from childhood and accompany the individuals to adulthood. Some of these determinants might be modified across individuals’ lifespan, affecting their personal food choices. The present findings could be applied as a guide to design appropriate strategies to improve food choices and dietary behaviors of Iranians.
